# MicroRNAs in metabolic effects with atypical antipsychotics—a scoping review

**DOI:** 10.1177/20451253261430603

**Published:** 2026-03-31

**Authors:** Weng Tong Wu, Deonna Setiawan, Stephen J. Glatt, Jen-Tsan Chi, Ping-I. Lin

**Affiliations:** Discipline of Psychiatry and Mental Health, University of New South Wales Sydney, Sydney, NSW, Australia; School of Medicine, Western Sydney University, Sydney, NSW, Australia; Department of Psychiatry, SUNY Upstate Medical University, Syracuse, NY, USA; Department of Molecular Genetics and Microbiology, Duke University, Durham, NC, USA; Discipline of Psychiatry and Mental Health, University of New South Wales Sydney, AGSM Level 3, Sydney, NSW 2052, Australia; Department of Psychiatry and Behavioral Neuroscience, Saint Louis University, St. Louis, MO, USA

**Keywords:** atypical antipsychotics, metabolic effect, microRNA, schizophrenia

## Abstract

**Background:**

Metabolic side effects associated with atypical antipsychotics represent a major challenge in the clinical management of schizophrenia, contributing to poor treatment adherence and an increased risk of relapse. MicroRNAs (miRNAs) have emerged as promising diagnostic biomarkers for schizophrenia, with growing evidence indicating that their expression is modulated by antipsychotic treatment. Dysregulated miRNAs may not only reflect underlying disease mechanisms but also actively contribute to therapeutic response and the development of metabolic side effects.

**Objectives:**

This scoping review critically evaluates the current literature on miRNAs in schizophrenia, focusing on their role in modulating treatment response and antipsychotic-induced metabolic disturbances. Key knowledge gaps are identified to inform future translational research.

**Eligibility criteria:**

We included studies involving adults or animal models with psychotic symptoms (with schizophrenia as the primary diagnosis) treated with atypical antipsychotics. Eligible studies reported associations between miRNA expression, metabolic parameters, and clinical outcomes.

**Sources of evidence:**

A rapid review was conducted using PubMed to identify relevant articles published up to December 1, 2025 and 16 articles were included for final review.

**Charting methods:**

Data charting was performed by one reviewer using a pre-developed and piloted form. The review was reported according to the Preferred Reporting items for Systematic reviews and Meta-Analyses extension for Scoping Reviews (PRISMA-ScR checklist).

**Results:**

Atypical antipsychotics, particularly those acting on dopamine and serotonin receptors, were shown to modulate specific dysregulated miRNAs. Several of these miRNAs regulate genes involved in metabolic pathways, such as lipid and glucose metabolism, potentially contributing to the variability in cardiometabolic side effects observed across individuals.

**Conclusion:**

Emerging evidence suggests that miRNAs may play a dual role in mediating both therapeutic efficacy and metabolic risk in schizophrenia treatment. However, the underlying mechanisms remain incompletely understood. Robust, large-scale studies are urgently needed to validate miRNAs as clinically actionable biomarkers for guiding personalized antipsychotic therapy.

**Trial registration:**

A protocol was not prospectively registered, as the aim of this scoping review was exploratory in nature.

## Plain language summary

How MicroRNAs may help explain metabolic side effects of common antipsychotic medications: a review

This review looks at small molecules in the body called microRNAs (miRNAs), which may help explain why some people with schizophrenia respond differently to antipsychotic medications and why these drugs often cause side effects like weight gain, high cholesterol, or high blood sugar. Researchers already know that miRNAs can act as biological markers for schizophrenia and that their levels can change after taking antipsychotic medications. This review focused on studies involving adults with psychotic symptoms who were treated with atypical antipsychotics (a common type of medication for schizophrenia). The findings suggest that these medications may help “reset” certain miRNAs that were off balance in people with schizophrenia. However, some miRNAs also control how the body processes drugs and fats, which may explain the wide range of metabolic side effects seen in patients. In short, miRNAs might be a key link between treatment response and side effects, but more research is needed to understand their exact role. This could one day help doctors personalize treatment and reduce harmful side effects.

## Introduction

### Challenges for clinical management of schizophrenia

Morbidity and mortality are critical concerns for individuals with schizophrenia, a severe mental disorder characterized by psychotic symptoms that significantly impair functional capacity. Accumulating evidence indicates that schizophrenia is associated with a mean life expectancy approximately 15 years shorter than that of the general population,^1^ a gap partially attributable to cardiovascular diseases.^2^ Metabolic syndrome—a constellation of central obesity, dyslipidaemia, glucose intolerance, and hypertension^3,4^—is found in approximately 33.5% patients with schizophrenia,^3^ though prevalence varied widely across different countries and studies.^4^ This susceptibility may be linked to the pathological effects on the serotonin receptor gene *5HT2A*, which influences both lipid levels and glucose tolerance.^5^ Furthermore, adverse metabolic effects associated with antipsychotics can further alter lipid metabolism,^6^ thereby increasing tcardiometabolic risk.^7,8^ These risks are often compounded by increased food cravings, subsequent higher calorie intake, and marked weight gain.^9^

Currently, second-generation antipsychotics (SGAs) remain the mostly widely prescribed medications for schizophrenia.^10^ Approximately 50% of patients receiving antipsychotics develop metabolic complications, with higher rates observing in young, first-episode patients^7^ and those prescribed SGAs rather than first-generation (typical) antipsychotics.^6^ Among SGAs, clozapine and olanzapine demonstrate the highest efficacy in treating psychotic symptoms;^2,11,12^ however, they also carry the greatest risk for weight gain,^13^ hyperglycemia and hyperlipidaemia.^4,14^ Aripiprazole also carries risks for hyperlipidemia and weight gain, albeit to a lesser extent, and is less frequently associated with increased blood glucose.^15^ Ultimately, these drug-induced metabolic dysfunctions contribute to the overall morbidity and mortality of this population.^16^ Hence, a deeper understanding of the mechanisms underlying SGA-attributed metabolic effects is imperative.

Against this backdrop, there is an urgent need to synthesize emerging evidence on molecular regulators that may explain both disease heterogeneity and treatment-related metabolic risk in schizophrenia. In particular, identifying novel biomarkers that illuminate previously unrecognized molecular mechanisms has the potential to inform new targets for intervention, improve risk stratification, and advance precision approaches to antipsychotic treatment. This review addresses this gap by focusing on microRNAs as candidate biomarkers linking antipsychotic exposure, metabolic dysregulation, and underlying pathophysiology.

### Association between miRNAs and schizophrenia

Emerging evidence suggests that novel biomarkers, such as microRNAs (miRNAs) may play a role in the pathophysiology of schizophrenia.^17^ Epigenetic mechanisms involving non-coding RNA can be categorized into long non-coding and small non-coding RNAs.^18^ A prominent example of the latter is miRNA, first discovered in *Caenorhabditis elegans* through genetic approaches—a breakthrough recognized with the 2024 Nobel Prize in Physiology or Medicine. In addition to regulating non-protein-coding transcripts, miRNAs modulate the expression of protein-coding genes, with their levels often correlating closely with gene function.^19,20^ They are abundant in the nervous system and play vital roles in neuroplasticity and neuronal functions, such as neurite outgrowth and synaptic signaling.^21^ These epigenetic mechanisms may influence biological functions,^22^ neurotransmission,^23^ and cognition.^24^ Given that psychosis is a subjective experience rooted in the brain, a largely inaccessible organ in research, researchers have pursued peripheral biomarkers. Notably, Noto et al.^25^ demonstrated that miRNA levels are linked to the pathophysiology of first-episode psychosis.

### Association between miRNA and antipsychotics

Prior work suggested that miRNAs may be involved in the mechanism of action of antipsychotics in bipolar mania.^21^ Previous studies have shown that antipsychotics can normalize the dysregulation of certain disease-related miRNAs.^26^ More recently, miRNA has been suggested as a promising biomarker for the diagnosis, management, and pharmacological monitoring of psychosis.^27^ As patients who exhibit significant increases in leptin, insulin, and C-peptide during the first 6 weeks of antipsychotics treatment are at higher risk for later relapse,^28^ it is possible that miRNAs contribute to heterogeneous drug responses by modulating gene expressions that impact the concentration of these biomarkers.^29^

Therefore, it is crucial to clarify how antipsychotics influence metabolism through miRNA expression.^16^ Researching these biomarkers could support early disease detection,^20^ enable patient-tailored treatment, and prevent the “trial and error” approach that often leads to non-adherence or relapse.^20^ Preliminary evidence also indicates that miRNAs can be used in cardiovascular cells for functional screening,^30^ which is particularly relevant given the cardiovascular burden in this population. Identifying miRNA dysregulation may also reveal new pharmaceutical targets,^30^ as miRNAs represent both an important intermediate target of drug action and a point of influence for disease-associated changes including these metabolic side effects.^13^

### Current knowledge

While experimental evidence regarding the role of miRNAs in regulating pharmacogenomics and drug responses is emerging, studies specifically addressing how miRNAs affect patient outcomes following antipsychotic treatment remain limited.^16^ This gap is significant, as metabolic side effects directly impact treatment adherence. Furthermore, there is growing recognition of the role of genomic regulation in antipsychotic response.^31^

This scoping review examines the current literature on the role of miRNAs in mediating the metabolic effects of antipsychotic medications in individuals with psychotic symptoms. While psychosis is a leading cause of global disease burden,^25^ schizophrenia represents the most extensively studied disorder within this spectrum. Consequently, this review focuses primarily on schizophrenia to ensure depth, consistency, and clinical relevance. By doing so, we aim to clarify how miRNA dysregulation contributes to the heterogeneity of metabolic side effects and to identify opportunities for biomarker-guided interventions. Given the breadth of study designs and outcomes in the field, a scoping review was chosen to be an exploratory methodology to map the heterogeneous and emerging evidence on miRNAs in antipsychotic treatment.

## Methodology

We searched the English language literature, from undefined start date (as shown in PubMed the earliest paper is in February 2009) up to 1, December 2025, using PubMed, crossing the keywords “metabolic,” “weight gain,” “blood pressure,” “glucose,” and “lipid” respectively and in turn with both “microRNA” and “antipsychotic.” There was no PROSPERO registration as it is not compulsory for scoping review, and no review protocol was published previously. Author (WTW) first carried out title and abstract screening, and then followed by full-text screening to determine eligible papers for the scoping review. Formal critical appraisal was not conducted, which aligns with standard scoping review methodology, due to the goal of mapping the breadth of evidence rather than assessing the study quality. The manuscripts identified were included in this scoping review after evaluating the quality of the research and relevancy to the various sections of this review based on the eligibility criteria. Authors of the identified manuscripts were contacted should additional sources were required. Definitions of mental illnesses were based on from the Diagnostic and Statistical Manual of Mental Disorders Fifth Edition, accessed on 30 November 2025. Papers were deemed *eligible* when they met these criteria: (1) had psychotic symptoms with schizophrenia as primary diagnosis as main population of investigation, (2) age range of participants was required to be equal to or greater than 18 and without upper age limit, (3) the intervention was identified as second-generation antipsychotics, equivalent to atypical antipsychotics, (4) using humans or animals as subjects of investigation, (5) papers written in English, (6) with full-text available, and (7) were published in journals. Papers were *excluded* if they met any of these criteria: (1) population without the aforementioned condition, (2) participants out of this age range, (3) medications other than second-generation antipsychotics, (4) article types such as protocol, systematic review, literature or narrative review, meta-analysis, abstracts or papers without full-text, conference papers, (5) with non-pharmacological interventions, and (6) articles not in English language. Data were extracted manually into a pre-developed and piloted form in Microsoft Excel application by two reviewers collaboratively (WTW, DS), and queries were discussed and resolved with another author (DL). The data was then summarized and further being created into a visual presentation to ease the understanding. With this study as a scoping review, we mainly reported the significance, including changes in miRNA in people with schizophrenia, impacts on miRNAs for those on second-generation antipsychotics as well as how it can further impact the metabolic profiles, which was declared by the authors in the manuscripts. The reporting of this study conforms to the PRISMA-scoping review guideline in the EQUATOR Network website, and a PRISMA-scoping review checklist^32^ is shown in Supplemental Table 1.

## Results

**Figure 1.**
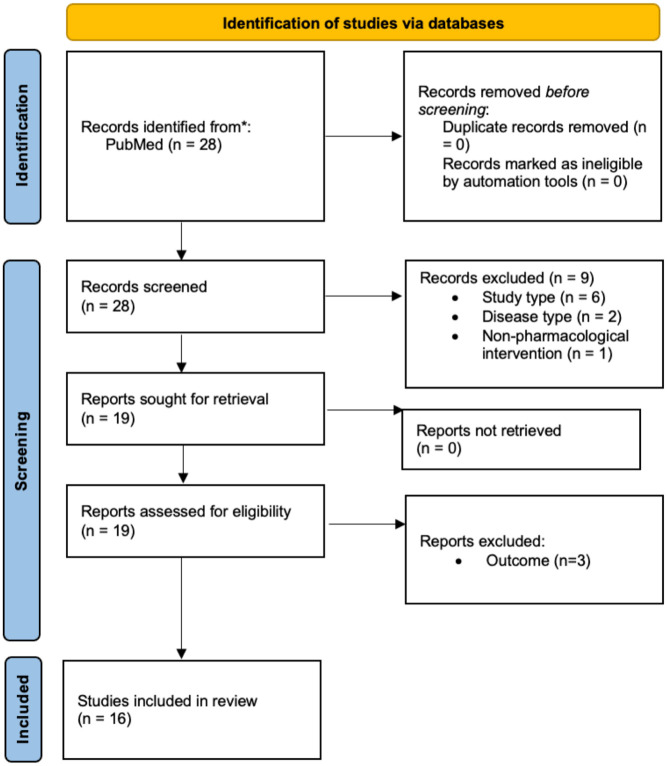
Preferred Reporting Items for Systematic reviews and Meta-Analyses (PRISMA) flow diagram for the current scoping review.

There were 28 papers identified from the initial search (full search string available as Supplemental File), where 9 papers were excluded after title and abstract screening, and the remaining 19 papers were screened for full-text, and 16 papers were deemed eligible for data retrieval (as described in Figure 1). Most of the studies focused on the changes of miRNA in schizophrenia, with less findings reported about the changes in expression level following atypical antipsychotic uses (demonstrated in Table 1), as well as the changes in miRNA post treatment (demonstrated in Table 2), treatment efficacy and response prediction, and lastly, the role of miRNA in metabolic changes post treatment (demonstrated in Table 3). There was conflicting evidence found in current literature evidence, and more research is needed in future. Overall, shifts in antipsychotic-induced miRNA levels serve as a molecular markers of efficacy, particularly miR-181b and miR-132, hold evidence for predicting treatment response and monitoring the metabolic side effects common in this population.

**Table 1.** Changes of miRNA in schizophrenia.

Author	Year	Subject (human/animal)	Significant miRNA changes in schizophrenia
Mellios et al.^33^	2009	Postmortem human brain samples (20 schizophrenia, 20 controls); adult male C57BL/6 mice	In human samples, miR‑195 levels did not differ significantly from controls; however, a subset of patients showed reduced mature miR‑195. No association was observed for miR‑30a‑5p. In BDNF‑deficient mice, miR‑195 was unchanged, while miR‑30a‑5p expression was increased.
Kocerha et al.^41^	2009	Adult male C57BL/6J mice treated with dizocilpine; NR1 mutant mice	Acute dizocilpine administration induced a reduction in miR‑219 expression in the prefrontal cortex at 15 min, which returned to baseline by 120 min. No changes were observed in the hippocampus or cerebellum. Chronic treatment did not alter miR‑219 levels. NR1 mutant mice exhibited reduced miR‑219 expression in both the prefrontal cortex and hippocampus.
Mellios et al.^34^	2012	Postmortem prefrontal and parietal cortex samples from schizophrenia patients and controls	Reduced levels of mature miR‑30b were observed in female patients with schizophrenia compared with female controls. No differences were detected in precursor forms of miR‑30b or in male samples.
Santarelli et al.^13^	2013	Plasma samples from 20 schizophrenia patients and 20 healthy controls	Plasma levels of miR‑181b, miR‑30e, miR‑34a, and miR‑7 were increased in patients with schizophrenia relative to controls.
Gardiner et al.^6^	2014	Plasma samples from 61 schizophrenia patients and 62 healthy controls	miR‑181b, miR‑30e, miR‑346, miR‑34a, and miR‑7 were upregulated in schizophrenia. miR‑132, miR‑195, miR‑212, and miR‑432 did not differ significantly between groups. Among all tested microRNAs, miR‑181b showed the strongest association with schizophrenia status.
Song et al.^35^	2014	Peripheral blood mononuclear cells from schizophrenia patients and controls; MK‑801-treated rat model	Multiple microRNAs, including miR‑132, miR‑664*, miR‑1271, miR‑200c, miR‑432, and miR‑134, were reduced in schizophrenia patients. miR‑132 was also reduced in both peripheral blood cells and whole brain tissue in the schizophrenia‑like rat model.
Sun et al.^36^	2015	Peripheral blood mononuclear cells from schizophrenia patients and controls	Microarray analysis identified widespread upregulation of multiple microRNAs, including miR‑1273d, miR‑1303, and miR‑21. Quantitative RT‑PCR confirmed upregulation of selected targets, while miR‑4701‑3p showed reduced expression.
Yu et al.^37^	2015	Prefrontal cortex of NRG1 and DISC1 mutant mice	miR‑29b expression was increased in heterozygous DISC1 mutant mice compared with wild‑type and homozygous mutants.
Chen et al.^38^	2016	Peripheral blood mononuclear cells from schizophrenia patients and healthy controls	No significant differences in miR‑195 expression were detected between schizophrenia patients and controls, including after adjustment for age and body mass index.
O'Tuathaigh et al.^42^	2017	Blood samples from first‑episode schizophrenia patients and healthy controls	miR‑203a‑3p expression was increased approximately fourfold in blood and blood‑derived exosomes from schizophrenia patients compared with controls.
Huang et al.^29^	2021	Postmortem prefrontal cortex RNA‑sequencing data (CommonMind Consortium)	A significant three‑way interaction was observed among schizophrenia risk, miR‑137 target genes, and co‑expression modules enriched for synaptic and neuronal processes.
Tsoporis et al.^39^	2022	Postmortem human brain samples (20 schizophrenia, 20 controls); adult male C57BL/6 mice	In human samples, miR‑195 levels did not differ significantly from controls; however, a subset of patients showed reduced mature miR‑195. No association was observed for miR‑30a‑5p. In BDNF‑deficient mice, miR‑195 was unchanged, while miR‑30a‑5p expression was increased.
Pergola et al.^40^	2024	Adult male C57BL/6J mice treated with dizocilpine; NR1 mutant mice	Acute dizocilpine administration induced a reduction in miR‑219 expression in the prefrontal cortex at 15 min, which returned to baseline by 120 min. No changes were observed in the hippocampus or cerebellum. Chronic treatment did not alter miR‑219 levels. NR1 mutant mice exhibited reduced miR‑219 expression in both the prefrontal cortex and hippocampus.

Articles sorted as per year of publication. miRNA*: antisense sequence to the mature miRNA.

miRNA, microRNAs.

**Table 2.** Changes in miRNA before and after antipsychotic treatment.

Author	Year	Subject	Antipsychotics	Changes in miRNA
Mellios et al.^33^	2009	Adult male C57BL/6 mice	Clozapine (5 mg/kg for 21 days)	No significant changes were observed in cortical miR‑195 or miR‑30a‑5p expression.
Kocerha et al.^41^	2009	Adult male C57BL/6J NR 1 mice on dizocilpine and another cohort with NR1 mutant	Clozapine (30 min before dizocilpine of 0.75 mg/kg)	Clozapine pretreatment prevented the dizocilpine‑induced reduction of miR‑219 expression in the prefrontal cortex.
Mellios et al.^34^	2012	Adult male C57BL/6 mice of 10–15 weeks of age, and human sample: 30 postmortem samples of PFC with schizophrenia and 30 PFC from control	Clozapine (21 days for 5 mg/kg)	Olanzapine treatment was associated with reduced expression of miR‑193, while clozapine treatment altered expression of miR‑329 and miR‑342‑5p.
Santarelli et al.^13^	2013	Whole brain sample of 76 male C57BL/6 mice of 8–10 weeks of age	Clozapine (10 mg/kg for 7 days), olanzapine (10 mg/kg for 7 days), and 0.9% saline	miR‑181b levels were reduced following treatment with all four antipsychotic medications, while other measured microRNAs remained unchanged.
Gardiner et al.^6^	2014	JM-Jurkat T-lymphocyte cells of humans with schizophrenia and non-psychiatric controls	Clozapine (400 ng/ml for 15 days)	Plasma levels of miR‑132 decreased after three and six weeks of treatment. miR‑181b, miR‑30e, and miR‑432 decreased after 6 weeks, with olanzapine producing the largest overall effect.
Song et al.^35^	2014	Plasma from 20 schizophrenia patients (11 males and 9 females, aged 18–56 (30.90 ± 11.94)) and 20 healthy control (12 males and 8 females, aged 18–58 (30.85 ± 12.10))	6 weeks of: Olanzapine (*n* = 5; dosage range 5–20 mg), quetiapine (*n* = 5, dosage range 100–800 mg), ziprasidone (*n* = 5, dosage range 40–140 mg), risperidone (*n* = 5, dosage range 2–6 mg)	miR‑132 expression increased following treatment, while miR‑1271 and miR‑664* showed moderate increases. Other microRNAs did not change significantly.
Sun et al.^36^	2015	Plasma from 61 schizophrenia patients and 62 healthy control -> 25 out of 61 SZ patients with Positive and Negative Syndrome Scale (PANSS) > 60 had medication intervention	3 weeks and 6 weeks of: Olanzapine (*n* = 25, dosage range 5–20 mg), quetiapine (*n* = 8, dosage range 100–800 mg), ziprasidone (*n* = 6, dosage range 40–140 mg), risperidone (*n* = 4, dosage range 2–6 mg)	miR‑21 expression decreased following treatment, whereas other examined microRNAs did not show significant changes.
Yu et al.^37^	2015	PBMC of 105 patients with SCZ (50 males and 55 females; mean age: 25.03 ± 8.34 years) and 130 healthy controls (60 males and 70 females; mean age: 22.73 ± 6.79 years) -first-onset schizophrenia and Chinese of Han descent were selected first -further *exclude those with increase in body weight of more than 10%*, serious side effects, or no significant improvement in 8 weeks After screening, only 10 patients maintained and fulfilled the monotherapy regimen and achieved remission.	Risperidone for 8 weeks, dosage range 3–6 mg/day	Clozapine treatment increased miR‑132 expression in the medial prefrontal cortex.
Chen et al.^38^	2016	PBMC from 82 SCZ (42 females 40 males; mean age 21.7 ± 4.09 years) and 43 healthy control (23 females 20 males; mean age 22.1 ± 4.00 years) Chose 30 SCZ patients for medication treatment	6 weeks: olanzapine (*n* = 10, dosage range 5–20 mg), quetiapine (*n* = 8, dosage range 100–800 mg), ziprasidone (*n* = 7, dosage range 40–140 mg), risperidone (*n* = 5, dosage range 2–6 mg)	miR‑195 expression decreased following treatment, with significant reductions observed in clinical responders but not in non‑responders.
O'Tuathaigh et al.^42^	2017	—	—	Olanzapine treatment attenuated, but did not fully normalize, the elevated expression of miR‑203a‑3p in blood and exosomes.
Johnstone et al.^43^	2018	mPFC of 11-week-old C57BL/6 mice (4 males, 4 females)	21 days with Clozapine (5 mg/kg) versus saline control	Clozapine induced dose‑dependent upregulation of cortical miR‑124‑3p, with no significant changes observed for miR‑124‑5p.
Huang et al.^29^	2021	PBMC of schizophrenia patients (*n* = 81, 45 males 36 females; mean age 25.01 ± 12.22 years) and healthy control (*n* = 93, 43 males 50 females; mean age 22.16 ± 4.71 years) 25 refused, 2 quality issues -> *n* = 52	2 months of Olanzapine (19.7 ± 4.85 mg/day)	Clozapine pretreatment prevented the dizocilpine‑induced reduction of miR‑219 expression in the prefrontal cortex.
Tsoporis et al.^39^	2022	Blood sample of first episode of SZ (*n* = 11, 6 males 5 females; mean age 24.5 ± 3.9 years) and healthy control (*n* = 10, 5 males 5 females; mean age 26.8 ± 4.5 years)	6 weeks of olanzapine -> 15 mg (*n* = 5), 20 mg (*n* = 6)	Olanzapine treatment was associated with reduced expression of miR‑193, while clozapine treatment altered expression of miR‑329 and miR‑342‑5p.
Swathy et al.^16^	2022	HepG2 (human liver cell line)	24 h of Clozapine (25 µM) vs control	miR‑181b levels were reduced following treatment with all four antipsychotic medications, while other measured microRNAs remained unchanged.
Pergola et al.^40^	2024	—	—	Plasma levels of miR‑132 decreased after 3 and 6 weeks of treatment. miR‑181b, miR‑30e, and miR‑432 decreased after 6 weeks, with olanzapine producing the largest overall effect.
Younis et al.^31^	2025	Six adult male C57b/6J mice about 8 weeks of age	Clozapine (21 days of 4 mg/kg/day) vs saline as control	miR‑132 expression increased following treatment, while miR‑1271 and miR‑664* showed moderate increases. Other microRNAs did not change significantly.

Articles sorted as per year of publication. miRNA*: antisense sequence to the mature miRNA.

miRNA, microRNAs.

**Table 3.** Metabolic side effects or treatment efficacy associated with antipsychotics.

Author	Year	Subject (human/animal)	Metabolic effects/behavioral changes
Kocerha et al.^41^	2009	Adult male C57BL/6J NR 1 mice on dizocilpine and another cohort with NR1 mutant	Mice receiving LNA–antimiR-219 (to inhibit miR-219) exhibited marked alterations in hyperlocomotion and stereotyped behaviors 30 min after dizocilpine administration compared with controls; these effects persisted for approximately 1 h.
Santarelli et al.^13^	2013	Whole brain sample of 76 male C57BL/6 mice of 8–10 weeks of age	*Clozapine*: Transcriptomic/pathway analyses highlighted weight gain–related metabolic terms (e.g., insulin resistance and obesity). Metabolic disease categories ranked among the top disease/disorder annotations, including metabolic and glucose metabolism disorders. Lipid metabolism ranked among the top molecular/cellular functions. *Olanzapine*: Nutritional disease categories ranked among the top disease/disorder annotations (e.g., nutritional disorder, obesity, and weight gain). Lipid and carbohydrate metabolism ranked among the top molecular/cellular functions. Metabolism-relevant pathways (including glucocorticoid receptor signaling and PPARα/RXRα activation) were reported in the olanzapine group.
Song et al.^35^	2014	Plasma from 20 schizophrenia patients (11 males and 9 females, aged 18– 56 (30.90 ± 11.94)) and 20 healthy control (12 males and 8 females, aged 18–58 (30.85 ± 12.10))	Change in miR-181b levels was positively correlated with improvement in negative symptoms and “lack of response” symptoms. Downregulation of miR-181b predicted 88.2% and 82.4% of treatment effects for negative symptoms and lack-of-response symptoms, respectively. For negative symptoms, the odds ratio for the higher treatment-effect subgroup (symptom score reduction rate ⩾ 50%) versus the lower treatment-effect subgroup (<50%) was 11.283. For lack-of-response symptoms, the corresponding odds ratio was 5.199. Receiver operating characteristic analyses indicated strong predictive performance for improvement in negative symptoms, whereas predictability for lack-of-response symptom improvement was not significant during antipsychotic treatment.
Sun et al.^36^	2015	Plasma from 61 schizophrenia patients and 62 healthy control -> 25 out of 61 SZ patients with PANSS > 60 had medication intervention	Plasma level changes in four microRNAs (miR-132, miR-181b, miR-212, and miR-30e) were strongly correlated with changes in PANSS scores after treatment. Decreases in plasma miR-132 and miR-432 were significantly larger in the high-effect subgroup (score reduction rate > 0.5) than in the low-effect subgroup (<0.5) after a 6-week treatment course.
Chen et al.^38^	2016	PBMC from 82 SCZ (42 females 40 males; mean age 21.7 ± 4.09 years) and 43 healthy control (23 females 20 males; mean age 22.1 ± 4.00 years) Chose 30 SCZ patients for medication treatment	Baseline miR-195 was not significantly associated with baseline PANSS total score or subscale scores (positive, negative, or general psychopathology). Baseline miR-195 was positively associated with the reduction rate in PANSS total score and the general psychopathology subscale. The reduction rate of miR-195 after treatment was also positively associated with reduction rates in PANSS total score and general psychopathology. Baseline miR-195 and age jointly predicted PANSS total score reduction rate (11.6% variance explained); baseline miR-195 predicted reduction in the general psychopathology subscale (6.1% variance explained). When miR-195 reduction rate was used as the predictor, no significant association was observed with PANSS reduction rates in regression models. Baseline miR-195 was reported as a significant predictor of olanzapine response.
Huang et al.^29^	2021	PBMC of schizophrenia patients (*n* = 81, 45 males 36 females; mean age 25.01 ± 12.22 years) and healthy control (*n* = 93, 43 males 50 females; mean age 22.16 ± 4.71 years)	Polygenic risk scores related to miR-137 were not associated with symptom levels; however, improvement in negative symptoms was inversely associated with the miR-137 polygenic co-expression index. Overall, higher expression of the “darkorange” gene module was associated with less improvement in negative symptoms following short-term antipsychotic treatment.
Tsoporis et al.^39^	2022	Blood sample of first episode of SZ (*n* = 11, 6 males 5 females; mean age 24.5 ± 3.9 years) and healthy control (*n* = 10, 5 males 5 females; mean age 26.8 ± 4.5 years)	Change in miR-181b levels was positively correlated with improvement in negative symptoms and “lack of response” symptoms. Downregulation of miR-181b predicted 88.2% and 82.4% of treatment effects for negative symptoms and lack-of-response symptoms, respectively. For negative symptoms, the odds ratio for the higher treatment-effect subgroup (symptom score reduction rate ⩾ 50%) versus the lower treatment-effect subgroup (<50%) was 11.283. For lack-of-response symptoms, the corresponding odds ratio was 5.199. Receiver operating characteristic analyses indicated strong predictive performance for improvement in negative symptoms, whereas predictability for lack-of-response symptom improvement was not significant during antipsychotic treatment.
Swathy et al.^16^	2022	HepG2 (human liver cell line)	Plasma level changes in four microRNAs (miR-132, miR-181b, miR-212, and miR-30e) were strongly correlated with changes in PANSS scores after treatment. Decreases in plasma miR-132 and miR-432 were significantly larger in the high-effect subgroup (score reduction rate > 0.5) than in the low-effect subgroup (<0.5) after a 6-week treatment course.
Pergola et al.^40^	2024	Data from three independent clinical cohorts: CATIE (Clinical Antipsychotic Trials of Intervention Effectiveness) Project (*n* = 249), University of Bari Aldo Moro cohort in UK Biobank sample (*n* = 72), and the University of Brescia (*n* = 107) *All three studies had patients with atypical antipsychotics	Changes in miR-21 were negatively correlated with improvement in PANSS positive symptoms, general psychopathology, and aggressiveness. miR-21 expression decreased more strongly in patients treated with olanzapine compared with other antipsychotic groups. In regression analyses, aggressiveness symptom change, together with miR-21 downregulation, accounted for 16.3% of the variance.

Articles sorted as per year of publication. miRNA*: antisense sequence to the mature miRNA.

miRNA, microRNAs.

### Effects of miRNA on schizophrenia

From the 16 included studies, both human and animal samples were used. There were various biomarkers reported to have significant changes and can potentially in biomarker of schizophrenia in future.

Comparing the postmortem brain samples of schizophrenia with controls, Mellios et al.^33^ showed a significant decrease only in mature miR-195 in subset analysis but not in miR-195. Mellios^33^ explained that the changes in NPY and SST mRNA in schizophrenia could be partly attributed to a negative regulatory effect of miR-195 on prefrontal BDNF protein levels. As Mellios^33^ concluded that BDNF could be more involved in the feedback regulatory loops with members of miR-30 family than miR-195, Mellios et al.^34^ later used postmortem samples again and showed that mature miR-30b was significantly reduced in females but not in males, which could be related to Esr1 SNP genotyping and then associated with age of onset of schizophrenia. Song et al.^35^ then used plasma from living schizophrenia patients and found a significant overexpression in four biomarkers different from previous studies: miR-181b, miR-30e, miR-34a, and miR-7. Similarly, Sun et al.^36^ who also a similar research methodology had the same finding as Song et al.^35^ This further indicated that miR-181b had a stronger association with schizophrenia patients and suggested that it would be a strong independent predictor for schizophrenia. Sun also reported a higher increase in miR-30e in lower age subgroup (age below 19) when compared to higher age subgroup (age above 37.5) schizophrenic patients. However, Sun et al.^36^ also indicated that miR-132, miR-195, miR-212, and miR-432 had no significant differences in between the samples, of which the second biomarker was similar to Mellios’s^33^ finding. Yu et al,^37^ Chen et al.^38^ and Huang et al.^29^ further used peripheral blood mononuclear cells from the plasma from living patients to examine the changes in miRNA in schizophrenia. Yu found that miR-132 and miR-432 were increased significantly, which was different from Sun’s finding.^36^ Yu further found that miR-664*, miR-1271, miR-200c and miR-134 to be significant reduced, which have not been reported in other studies. Yu suggested that the ROC analysis supported these six markers can also be used as unique marker for schizophrenia. Chen et al.^38^ further discussed 10 different biomarkers that were significantly increased after microarray expression testing followed by qRT-PCR. Of these biomarkers, miR-21, which was reported to have no significant difference in Yu’s^37^ study with miR-21*, was not further reported in other studies to further verify for the significant increase in living schizophrenia patients. Huang et al.^29^ further supported Mellios’^33^ and Sun’s^36^ findings of a non-significant difference in miR-195 expression levels. Tsoporis et al.^39^ and Pergola et al.^40^ further reported miR-203a-3p and miR-137 respectively be related to schizophrenia. Pergola^40^ explained that the co-expression of miR-137 inside darkorange gene (expressed in pre-frontal cortex during young adulthood and functionally enriched for synaptic signaling and nervous system development) was associated with emotion processing, and prefrontal neuronal functioning and maturation during adolescence, which is a critical period of development for the onset of psychopathology in schizophrenia.

For the animal samples, Mellios et al.^33^ found a significant increase in miR-30a-5p in BDNF- deficient C57BL/6J adult mice but not for miR-195. Kocerha et al,^41^ who also used C57BL/6J adult mice but used dizocilpine (phenycyclidine-like NMDA-R antagonist) to rapidly produce schizophrenia-like behavioral deficits, found a reduction of miR-219 in tissues from pre-frontal cortex, hippocampus, and cerebellum after 15 min of injection. Kocerha^41^ proposed that miR-219 may have a role in regulating NDMA-R functioning and was altered during states of NMDA-R hypofunction, further causing schizophrenia-like symptoms. Similar finding was also reported in NR1 mutant mice in Kocerha’s paper, of which the first two brain regions are well implicated in the presentation of schizophrenia-related symptoms.^41^ However, the expression level in prefrontal cortex returned to normal after 120 min and no significant changes in those treated with 5 days of dizocilpine injection, which Kocerha^41^ explained as the latter finding to be related to desensitization over time. In Yu et al.^37^ rats treated with MK-801, which have been found to exhibit schizophrenia-like symptoms, were found to have a significant reduction in miR-132 in the whole brain tissue and peripheral blood mononuclear cells. O'Tuathaigh et al.^42^ further reported another biomarker, miR-29b to have a significant upregulation in prefrontal cortex of adult HET mice.

### Effects of antipsychotics on miRNAs

Olanzapine, risperidone, quetiapine, and ziprasidone were used in the studies of human and animal tissues. The expression level of miR-181b but not miR-132, miR-195, miR-212, miR-30e, miR-346, miR-34a, miR-432, and miR-7 was significantly downregulated in all four drugs as shown in plasma samples from Song et al.^35^ Chen et al.^38^ who also used same methodology, instead reported a significant reduction in miR-21 and different non-significant biomarkers. However, Sun et al.^36^ who used similar methodology but further classified the duration into 3 and 6 weeks respectively, observed a significant decrease in miR-132 in 3 and 6 weeks but miR-181b, miR-30e and miR-432 only in 6 weeks after medication use. Sun^36^ also reported olanzapine had the strongest effect on changes of plasma miRNA expression levels. Sun further compared these patients with other schizophrenia patients on different psychotropic medications, reporting all except miR-181b, and adding miR-195 to be significantly lower in plasma level.

For olanzapine, Santarelli et al.^13^ reported a significant decrease in miR-193 (predicted target associated with HTR1A) whereas Huang et al.^29^ found a decrease in miR-195 in the treatment responders (based on the PANSS score). Tsoporis et al.^39^ instead reported that the upregulation of miR-203a-3p in blood exosomes induced by schizophrenia was prevented by olanzapine, but the correlation between pre-and post-therapy measurements was weak.

For risperidone, different to Song’s^35^ and Sun’s^36^ findings, Yu et al.^37^ reported a upregulation of miR-132 and also observed a moderate significantly increase in miR-1271 and miR-664*. Whilst Yu^37^ reported no significant changes in miR-134, miR-200c, and miR-432, the last biomarker was supported by Sun’s^36^ finding.

The effect of clozapine was also measured in several studies. For clozapine use in mice models, no significant changes were found in miR-195^33^ miR-30a-5p,^33^ miR-30b,^34^ and miR-124-5p.^31^ However, Santarelli et al.^13^ reported a significant decrease in miR-329 and miR-342-5p, predicted targets associated with HTR2C. Similar to Yu’s^37^ finding in risperidone, Johnstone et al.^43^ found a significant upregulation of miR-132 in clozapine use. Younis et al.^31^ further reported a significant upregulation in miR-124-1hg, weaker upregulation in miR124-2hg and a dose-dependent upregulation in mir-124-3p. As miR-124a-1hg is the dominant source of microRNA,^31^ the significant downregulation may explain the treatment effects observed in schizophrenia in combination with the changes in miRNA mentioned above. Kocerha^41^ further found that pretreatment with clozapine prevented a reduction of miR-219, which are highly sensitive to disruptions in NMDA-R signaling, in prefrontal cortex after administration of dizocilpine in mice. There were different biomarkers reported in human samples in clozapine use; there were no significant changes in miR-17-3p and miR-21-5p in the study with T-lymphocyte cells, but reported eight mRNA: miRNA pairs (where miRNA typically inhibits/destabilizes mRNA expression) and other molecules (Protein kinase interferon-inducible double-stranded RNA dependent activator and programmed cell death 10) to be upregulated after subacute treatment with clozapine.^6^ Swathy et al.^16^ further used human liver cell line HepG2 to show significant upregulation and downregulation of a range of other biomarkers with 24 h of clozapine use comparing to control.

### Treatment efficacy and prediction

Several studies suggest that miRNAs may be used to monitor treatment response. Kocerha et al.^41^ had reported a significant altered hyperlocomotion and stereotypy in animal samples with antipsychotics that inhibits miR-219, with an effect lasting for only an hour. Sun et al.^36^ reported that the changes in human plasma level of miR-132, miR-181b, miR-212, and miR-30e were highly correlated to the changes in the PANSS clinical scores in patients after antipsychotic medication, and the decrease in miR-132 and miR-432 was significantly greater in higher effect subgroup than the lower-effect subgroup classified by the PANSS score reduction rate after 6 weeks of treatment course. Chen et al.^38^ added that the change in miR-21 was significantly reduced in olanzapine than other atypical antipsychotics, of which the change was negatively correlated with the improvement of positive and aggressive symptoms alongside general psychopathology. Chen^38^ further found that 16.3% of the observed variance in downregulation of miR-21 can be statistically explained by the change in aggressiveness symptoms. Tsoporis et al.^39^ also reported that blood plasma or blood exosome expression level of miR-203a-3p had positive strong correlation with positive or negative PANSS scores after treatment measurements. Pergola et al.^40^ further added that the polygenic co-expression index of miR-137 (in darkorange gene) to be negatively associated with improvement in negative symptoms and concluded that a greater darkorange gene expression to be associated with less improvement in negative symptoms after short-term antipsychotic treatment. Song et al.^35^ reported the significant downregulation of miR-181b to be positively related to improvement of negative symptoms and lack of response symptoms, and suggested with a ROC curve that the downregulation has significantly great predictability of negative symptoms improvement along the antipsychotic treatment. Huang et al.^29^ further reported that both baseline and reduction rate of miR-195 were significantly positively associated with both the reduction rate of PANSS total and general psychopathological subscale score, concluding that baseline miR-195 expression level might be a significant predictive factor for olanzapine response. Overall, these findings suggest that antipsychotic-induced alterations in miRNA expression are not only associated with changes in clinical symptoms but may also serve as potential biomarkers for predicting and monitoring treatment response in patients with schizophrenia.

### Effects on metabolic profiles

For clozapine, Santarelli et al.^13^ reported lipid metabolism, insulin resistance, weight gain and obesity, and metabolic disorder and glucose metabolism disorder as the most significant side effects observed in mice. Axonal guidance signaling and metabolic pathways such as cellular movement, carbohydrate metabolism, and cellular growth and proliferation were impacted post antipsychotics use.^13^ Swathy et al.^16^ reported similar pharmacokinetic pathways being impacted in human samples after clozapine use alongside the changes of a list of miRNAs: ABC transporters, drug metabolism cytochrome P450, drug metabolism other enzymes and metabolic pathways, which may further lead to the observed side effects.

For olanzapine, similarly, nutritional disorder, obesity, weight gain, impaired lipid metabolism, and carbohydrate metabolism involving the glucocorticoid receptor signaling and PPARα/RXRα activation were reported.^13^ Gardiner et al.^6^ concluded that the antipsychotic-induced weight gain from increased accumulation of lipid and fatty acid to be associated with impaired oxidation of fatty acid and then dysregulating genes related to oxidative or cellular stress, including mitochondrial dysfunction, free radical scavenging, impaired permeability of mitochondrial membrane, and dysregulated quantity of hydrogen peroxide, NADPH, and reactive oxygen species.

### Recap of results

The review of 16 studies highlights several recurring miRNAs—miR-181b, miR-195, and miR-132—as key markers for schizophrenia pathology and treatment response. MiR-181b is consistently overexpressed in human plasma and is highly sensitive to antipsychotics, with significant downregulation that correlates with improved negative symptoms. miR-195 shows a split in central and peripheral levels—while it is reduced in postmortem brain tissue, it is often unchanged in baseline human plasma. Its reduction during treatment is a strong predictor of responses to olanzapine. MiR-132 exhibits conflicting baseline directions, where an increase in human peripheral blood was reported but a decrease in rat brain models. Nevertheless, it remains a consistent marker for monitoring PANSS clinical score changes.

There was heterogeneity in study samples, with evidence from human and animal tissues. The human studies primarily link miRNAs to clinical clusters. For example, miR-137 is correlated to adolescent prefrontal maturation, while miR-21 and miR-203a-3p are correlated with aggression and general psychopathology. On the other hand, animal or in vitro models provide mechanistic depth. While mouse models link miR-219 to NMDA-receptor hypofunction, cell-line studies show how clozapine-induced miRNA changes dysregulate metabolic pathways and drug-metabolizing enzymes.

## Discussion

Schizophrenia requires long-term, and potentially lifelong, medication management.^44^ While antipsychotic drugs are known to interfere with metabolic functions and induce severe metabolic side effects,^16^ a lack of insight resulting from cognitive impairment in schizophrenia may also contribute to non-adherence.^45^ As poor adherence often leads to diminished therapeutic responses, it remains the strongest predictor of relapse in schizophrenia. Furthermore, it increases suicidal risk, mortality rates, and overall functional impairment, ultimately worsening the clinical prognosis.^46^ Consequently, advances in pharmacological research should focus on the biological mechanisms underlying the adverse effects that drive poor treatment adherence.

### Clinical utility of miRNA in disease monitoring

Over the past decade, significant progress has been made in clinical studies investigating miRNAs as potential diagnostic and therapeutic targets.^30^ Due to their high stability in human plasma and tissue, miRNAs are valuable tools for diagnostic assessment,^47^ particularly when profiled using generalized sequence-based methods.^19^ It can be exemplified by Keller et al.^48^ who utilized miRNA profiling to accurately predict disease in more than two-thirds of study subjects across 14 distinct conditions. Similarly, Hydbring and Badalian-Very^49^ used miRNAs to distinguish effectively between types of heart disease, muscular disorders, and neurodegenerative conditions. Research by Richardson et al.^50^ also demonstrated that approximately 22% of single-nucleotide polymorphisms mapped to miRNAs are associated with specific disease phenotypes. Beyond the low costs and rapid processing times associated with next-generation sequencing,^49^ these phenotypic assays are straightforward and adaptable to most disease-relevant cellular contexts.^30^ Given the evidence of miRNA dysregulation across various pathologies and the accuracy of modern profiling techniques,^49^ miRNA-based diagnostics are poised to become efficient clinical diagnostic tool in the near future.^51^

### Therapeutic potential of miRNAs and precision medicine

Advancing miRNA diagnostics from experimental research into clinical practice holds promise for personalized medicine, where the objective is to link specific miRNA profiles to disease modulation while minimizing adverse effects.^49^ Keller et al.^48^ indicated that up to 60% of differences in observed miRNA profiles may stem from distinct hematopoietic lineages.^52^ These variations can facilitate the development of specific targets for cell-culture-based screening and patient- tailored interventions.^31^

**Figure 2.**
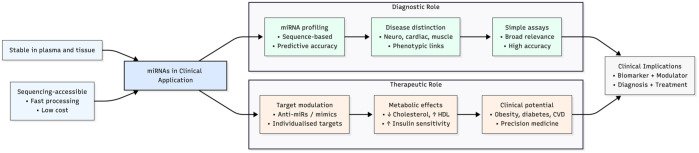
Overview of miRNAs in clinical application as diagnostic tools and therapeutic targets. This diagram illustrates the dual role of microRNAs (miRNAs) in modern clinical settings. Owing to their high stability in plasma and tissue and accessibility via low-cost, rapid sequencing, miRNAs are increasingly utilized in disease diagnostics—enabling accurate profiling, phenotypic discrimination, and broad disease coverage. Concurrently, miRNAs are emerging as therapeutic modulators, with evidence supporting the use of anti-miRs and mimics to target metabolic and cardiovascular disorders, such as obesity, diabetes, and dyslipidemia. These complementary roles highlight miRNAs’ bridging potential as both biomarkers and modulators, underscoring their relevance to precision medicine. miRNAs, microRNAs.

Practical applications are already emerging in preclinical models. For instance, chimpanzees treated with antagonists of specific miRNAs that regulate cholesterol metabolism exhibited a reduction in total serum cholesterol.^53^ Similarly, studies in non-human primates demonstrate that anti-miRNAs targeting regulators of fatty acid homeostasis lead to a reduction in very-low-density lipoprotein (VLDL) triglycerides and a concomitant increase in high-density lipoprotein (HDL).^54^ Furthermore, evidence suggests that treatment with specific anti-miRNAs can improve glucose homeostasis and insulin sensitivity,^55^ implying therapeutic utility of anti-miRNAs across various metabolic disorders, including obesity, type 2 diabetes, and hyperlipidaemia.^56^ Research also indicates the potential of miRNA mimics or inhibitors in both treating and preventing cardiovascular disease in mice.^30^ Overall, miRNAs may act as both biomarkers and modulators of treatment,^23,49,51^ making them a pivotal area of research for precision medicine.^30^ An overview of the roles of miRNAs is illustrated in Figure 2.

### Bridging the gap—from preclinical evidence to translational application

There is a critical distinction when comparing miRNAs with immediate translational applicability against those supported primarily by preclinical data. miRNAs such as miR-181b, miR-132, and miR-195 demonstrate high translational potential because their dysregulation has been consistently validated in human plasma and correlates directly with clinical symptom scales like the PANSS. These biomarkers obtained in peripheral samples are crucial for clinical practice as they are accessible via minimally invasive blood collection, making them viable for real-time treatment monitoring.

On the other hand, markers such as miR-219 and miR-30a-5p remain largely within the preclinical domain. While they have been successfully used in animal models to map specific pathways, such as NMDA-receptor hypofunction or BDNF deficiency, their evidence base is still limited to brain tissue or specific genetic mouse strains. While these preclinical findings are important for identifying future therapeutic targets, a validation in human longitudinal studies in future is required before they can be integrated into diagnostic protocols or to guide clinical management.

## Limitations

This review has shown preliminary findings of certain specific miRNAs that make have an impact in the responses on atypical antipsychotics in schizophrenia. However, there were several limitations that readers need to be cautious of. There is a potential for publication bias, as studies with positive or significant findings may more likely be published. The small sample sizes in the reviewed literature may increase the risk of type I errors and further limit the generalizability of the results to the broader population of individuals with schizophrenia. Also, the included studies had significant methodological variability, including tissue source (discrepancies between post mortem brain tissue and peripheral blood samples, participants being antipsychotics naïve or not), duration of treatment (variability in measuring miRNA shifts at 3 weeks versus 6 weeks or longer, duration and dosage of medication usage), extraction techniques (differences in miRNA isolation and sequencing platforms) and data analysis. Future study designs can incorporate these variables as covariates in their analysis to further assist readers to interpret the findings in between the heterogeneous studies. Furthermore, as the team only utilized PubMed/MEDLINE database for the review, it may have resulted in missing out on relevant studies. However, PubMed/MEDLINE is the most comprehensive database for biomedical literature, and our search was complemented by manual reference screening. Future studies can look into utilizing other genetic databases such as SZDB^57^ to retrieve data and verify if other insights were offered regarding the association. As there were still limited studies that examined such relationship and that none of the included studies have measured the association between the changes in expression level of miRNA and prevalence of side effects, more large-scale review studies and trials need to be carried out to further examine the role of miRNAs in the mechanism of the SGA as well as how the metabolism can impact the side effects in further impacting the adherence rate.

### Implications

Given these inconsistencies, these miRNAs should currently be viewed as emerging candidates rather than established clinical tools. Continued research with larger, standardized cohorts is essential to confirm their reliability before they can be integrated into routine psychiatric practice. Although we found no data on how variations in miRNAs can predict the prognostic outcomes such as determining the treatment adherence and resistance and limited data on treatment responsiveness, future studies may consider clinical trials in strengthening the role of miRNAs in clinical application. With further research determining the correlation between SGA and metabolic profile in addition to miRNAs in schizophrenia patients with psychotic symptoms, pharmacological products that target the post-antipsychotic effectors of miRNAs may help in reducing the discontinuation rates of antipsychotics and alleviating subsequent costs to the individuals, their family, and the community. This will further assist clinicians in decision-making for clinical management for people with psychotic symptoms as well as to reduce healthcare costs for outpatient visits and in-patient hospital length of stay.

## Conclusion

Schizophrenia is a chronic mental condition that may lead to long-term functional deconditioning and physical comorbidities. With metabolic side effects from atypical antipsychotics, medication non-compliance has been the major management challenge. Emerging evidence suggests that patients with schizophrenia exhibit distinct miRNA expression profiles, which may underlie the variability in treatment response and susceptibility to metabolic side effects associated with atypical antipsychotics. Understanding these miRNA-mediated mechanisms is not only essential for elucidating disease pathology but also holds significant promise for clinical translation. By identifying miRNAs as potential biomarkers or therapeutic targets, future research could pave the way for personalized treatment strategies—enhancing drug efficacy, reducing adverse effects, and improving long-term adherence. This scoping review highlights the potential of integrating miRNA profiling into clinical practice to advance precision psychiatry, particularly by enabling the customization of antipsychotic treatment based on individual biological profiles. These findings underscore the need for future research to further investigate these associations, with the aim of informing and potentially revising current clinical management guidelines.

## Supplemental Material

sj-docx-1-tpp-10.1177_20451253261430603 – Supplemental material for MicroRNAs in metabolic effects with atypical antipsychotics—a scoping review Supplemental material, sj-docx-1-tpp-10.1177_20451253261430603 for MicroRNAs in metabolic effects with atypical antipsychotics—a scoping review by Weng Tong Wu, Deonna Setiawan, Stephen J. Glatt, Jen-Tsan Chi and Ping-I. Lin in Therapeutic Advances in Psychopharmacology

## Declarations

**Ethics approval and consent to participate:** Not applicable.

**Consent for publication:** Not applicable.

**Availability of data and materials:** Not applicable.
